# Method of Equivalent Error as a Criterion of the Assessment of the Algorithms Used for Estimation of Synchrophasor Parameters Taken from the Power System

**DOI:** 10.3390/s24144619

**Published:** 2024-07-17

**Authors:** Malgorzata Binek, Pawel Rozga

**Affiliations:** Institute of Electrical Power Engineering, Lodz University of Technology, Stefanowskiego 20, 90-537 Lodz, Poland; malgorzata.binek@p.lodz.pl

**Keywords:** equivalent error, PMU, RBF, DFT, synchrophasors, phase and amplitude estimation

## Abstract

The development of digital techniques in control engineering leads to the creation of innovative algorithms for measuring specific parameters. In the field of electric power engineering these parameters may be amplitude, phase and frequency of voltage or current occurring in the analyzed electric grid. Thus, the algorithms mentioned, applied in relation to the quoted parameters, may provide precise and reliable measurement results in the electric grid as well as ensure better grid monitoring and security. Signal analysis regarding its identification due to the type of interference is very difficult because the multitude of information obtained is very large. In order to indicate the best method for determining errors in measuring synchronous parameters of the measured current or voltage waveforms, the authors propose in this paper a new form of one error for all testing functions, which is called an equivalent error. This error is determined for each error’s value defined in the applicable standards for each of selected 15 methods. The use of the equivalent error algorithm is very helpful in identifying a group of methods whose operation is satisfactory in terms of measurement accuracy for various types of disturbances (both in the steady state and in the dynamic state) that may occur in the power grid. The results are analyzed for phasor measurement unit (PMU) devices of class P (protection) and M (measurement).

## 1. Introduction

The power system consists of devices which are responsible for the generation, transmission, distribution, storage, and utilization of electricity. The main task the power system must ensure is supplying the energy to consumers in a continuous and uninterrupted manner, simultaneously minimizing the financial costs. Therefore, the power system must meet technical requirements that will ensure safe use and the required quality and reliability in the supply of electricity. Nevertheless, ensuring stability in energy supply forces the system supervision by measuring the technology providing network monitoring and real-time system status assessment [1]. The power system under operation is vulnerable to disturbances that may spread in a short time and cover a large area of the system. Therefore, their elimination usually involves identifying the place of their occurrence, the type of disturbance and selected parameters of the disturbance’s characteristic values. Continuous and reliable supervision over the operating status of the power system, in order to ensure power security, is carried out by many systems, including, first of all, the Wide Area Measurement System (WAMS), which has phasor measurement units (PMUs) located at selected points of the power system. At the point of installation, PMUs measure instantaneous values of current or voltage based on the measurements of the phasor angle, magnitude, frequency, and rate of change of frequency (ROCOF) [2]. The PMU devices are installed in the power stations, the choice of which depends on the availability of the measurements being able to be conducted in a given station. In most applications, phasor data are obtained from locations with large distances from the PMU, which is why communication connections and data concentrators play an important role in collecting the data. PMU devices are synchronized by GPS (Global Positioning System) usually at one-second intervals [2,3,4]. They can determine the instantaneous phase angle of the phasor as the angle of the phasor relative to the coordinate system rotating with the rated frequency of the power system, synchronized with the Universal Time Clock (UTC) from the GPS system and mark each estimated value with a time stamp from the GPS system. The quantities calculated and marked in this way are called synchrophasor parameters, while the measurements are called synchronous measurements. The synchronous measurements in the WAMS, performed under the supervision of GPS devices, generate a synchronization error resulting from the accuracy of the GPS system, which is approximately 1 μs. In the synchronous measurements, this error corresponds to an angle shift of 0.018° or 0.0314 rad, which approximately generates a measurement error of about 0.03% [5,6,7].

As was mentioned above, the sinusoidal nature of voltages and currents in electricity transmission in a dynamic power system causes significant distortions in voltage and current waveforms (including, for example, voltage or current fluctuations, waveform distortions, and increases in voltage or current values) [8,9,10,11]. Signal oscillations can contribute to the sudden load changes, switching phenomena in transmission lines and, finally, even the blackout event. Therefore, accurate measurement of currents and voltages with an appropriately fast response time ensures the proper functioning of the modern power system (even small oscillations resulting from the structure of the power system may lead to a wide area blackout [9]). Thus, an important task in terms of protection and control of the power system operation is to ensure reliable and precise measurements of network parameters and then analyze them in a proper, fast time manner. Without a doubt, the main criterion for this goal is the obtainment of a high measurement accuracy.

When it comes to the PMU devices, determining the phasor parameters is possible using various algorithms, wherein an innovative algorithms are most often used. Obviously, the applicable standards impose appropriate requirements in the form of permissible measurement error values of synchrophasor parameters for disturbances, taking into account the class P (Protection) and class M (Measurement) of PMU devices operation. This is performed to assess the effectiveness and suitability of the measurement algorithm used and to check its sensitivity to an interference in input signals in the form of higher harmonics, aperiodic components, out-of-band interferences, range of frequency, or changes of frequency. The possibility of using various measurement algorithms for estimation of the amplitude, angle, and frequency of the synchrophasor means that a large number of results are obtained during this task. The solutions associated with protection, control, and monitoring of electric power systems introduce a variety of the parameters, on the basis of which the above-mentioned research is realized [3].

The literature reports dealing with this problem are very extensive, because the multitude and diversity of solutions influence the choice of a calculation method that adapts to the selected task in a specific phasor application. Phasor parameters for PMU devices can be determined based on the use of different methods. The algorithms used in determining phasor parameters include, first of all, methods based on Fourier Transform (FT) [12,13,14,15,16,17,18,19,20], which are very popular due to the speed and accuracy of data processing and the simplicity of application. In determining the phasor parameters, the Monte Carlo (MC) method [21] is also applied in the analysis of real data. In turn, the Wavelet Transform (WT) method and the Hilbert Transform (HT) method are used to detect the disturbances: noise and harmonics [22]. In the literature, the Mean Squared Error (MSE) method can also be found to be suitable to solve the problem of determining the synchrophasor parameters, most often in the form of Weighted Least Squares (WLS) [23,24] or Recursive Least Square (RLS) in matrix calculations [25], the Phase Lock Loop (PLL) method [3,26], the Quadrature Filter (FQ) [1,5,6,7] or the Kalman Filter (KF) [27]. Currently, methods based on Artificial Neural Networks (ANNs) are becoming increasingly popular, including network models described already in 1987 by R. Lippmann: the Hopfield network or Hamming network [28], Convolutional Neural Network (CNN) used to identify events in extracts of complementary features [29], a Machine Learning (ML)-based method used for fault location [25], Radial Basic Function (RBF), Linear Neural Network (LNN), and Feedforward Neural Network (FNN), all presented in [1,2,3,4,9,10,11,30,31,32], or the physics-aware pruned neural network (P2N2), based on the topology of the physical network and used to design connections between different hidden layers of a neural network model in order to improve the accuracy of voltage estimation in the distribution system [30]. Methods combining classical algorithms with neural networks are gaining more and more popularity in analyzing phasor parameters. An example may be the Discrete Wavelet Transform (DWT) combined with Deep Neural Networks (DNNs). This is because they provide greater accuracy of calculations and quick feedback regarding the verification of a disturbance or its location in the power network [33]. As mentioned above, the measurement requirements have been specified in the standards for two different performance classes: class P and class M. The first one is used when the application requires a quick response, while the second one is used when the response speed is not critical and the measurement precision can be increased at its expense. Since the number of results to be analyzed is very large, choosing the correct algorithm to analyze them is a concern in this area. However, due to the fact that there are several dozen or even several hundreds of different algorithms which are able to be chosen, it may be difficult to analyze so many obtained data results.

Hence, in order to indicate a method that ensures the highest accuracy and reliability of measurement for many testing functions, mainly from the point of view of development of security technology and control process, the authors propose a new, innovative algorithm called the method of equivalent error. The first approach to present it was the authors’ earlier work [4], where information on how to determine synchrophasor errors and the permissible values were specified. Herein, the work is focused on methods that meet the requirements for measurement errors in relation to the values of permissible errors specified in the applicable standards. Thus, the paper compares equivalent errors determined for six error values defined in [5,6,7] depending on the type of testing function (for 106 or 182 test functions for the classes P and M, respectively) for each of the fifteen selected methods. In other words, the aim of the paper is to present the method, called by the authors the method of equivalent error, which is necessary when analyzing a large number of results obtained from the algorithms of estimation of the amplitude, phase, and frequency of the synchrophasor, e.g., by PMU devices in the WAMS. Specifically, as an original approach, which has not been proposed yet, the authors define one equivalent error for all static and dynamic test functions excluding those functions with a step magnitude or functions with a step phase (functions from 1 to 98 for class P and functions from 1 to 174 for class M) and one equivalent error only for functions with a step magnitude or functions with a step phase (functions from 99 to 106 for class P and functions from 175 to 182 for class M). Based on the above, two equivalent errors determine the total error equivalent to clearly indicate the method, which provides the smallest total error equivalent for all test functions (which correspond to a disturbance that may appear in the power system).

The organization of the paper is as follows. Section 2 presents the requirements im-posed on the estimation methods of phasor parameters of the signal model used as well as a new equivalent error algorithm. Section 3 presents the numerical results for the P and M class in relation to the analyzed population of the functions, while Section 4 discusses the simulation results. Conclusions are given in Section 5.

## 2. Methodology of the Studies

### 2.1. The Permissible Errors of Phasor Parameters

Phasor in relation to a current or a voltage signal is a vector rotating in the coordinate system with the electrical power system’s nominal frequency. In this regard, a synchrophasor is a phasor with a UTC timestamp in every sample [5,6,7].

Standards [5,6,7] define, in general, two types of signals of static and dynamic types applied for PMU devices of the P (protection) and M (measurement) class. As was mentioned above, class P is used in the area of protection that requires a quick response and simultaneously permits less precise measurements, particularly when the basic waveform of the voltage or current signal has a frequency different from 50 Hz. In turn, the M class devices are applied in the situation when the most important issue is measurement accuracy and when a longer delay than for the P class is tolerable. Different sequences of the testing functions are proposed for each of the above mentioned classes, which were described in detail in [4]. For all of these functions, the errors must be estimated by the PMU devices, as per IEEE Standards [5,6,7].

The testing functions implemented in the authors’ simulations refer to all types of disturbances described in [5,6,7] and were described in [4]. Additionally, for static and dynamic signals, IEEE Standards [5,6,7] impose requirements in the form of permissible errors. For the purposes of implementing their own method, called the equivalent error, the authors conducted appropriate tests that meet the requirements [5,6,7] of permissible errors for

(a) All testing functions excluding step functions:
  - Total vector error (TVE),
  - Frequency error (FE),
  - The rate of change of frequency error (RFE)
(b) All testing functions for step functions:
  - Response time of TVE (RT-TVE),
  - Delay time (DT),
  - Overshoot/Undershoot value (OV).

The definition of the above quantities (TVE, FE, RFE, RT-TVE, DT, OV) was presented in the authors’ earlier article [4].

Definitions of the synchrophasor errors according to Standards [5,6,7], as well as the method of analyses of synchrophasor parameters for each functions, are described in detail in [4].

Appropriate tests for the selected 15 synchrophasor parameter estimation methods, taking into account the limitations for the obtained errors specified in the standards [5,6,7], were carried out for one continuous signal successively containing all testing functions, where one testing function lasts 1 s of the waveform analyzed. The exceptions are dynamic functions with a positive and negative ramp frequency, where the frequency range is ±2 Hz for P class and the frequency changes (increases/decreases) for 4 s.

In addition, at the beginning of testing, a signal with a frequency value equal to the value of the initial frequency will be fed for 0.4 s, and a signal with a frequency value equal to the value of the final frequency will be given for 0.6 s at the end of testing. The total duration of the signal is 5 s.

However, in the case of the M class, where the frequency range is ±5 Hz, the frequency will change (increase/decrease) for 10 s. Additionally, at the beginning of the test, a signal with a frequency value equal to the initial frequency will be given for 0.4 s, and at the end of the test, for 0.6 s a signal with a frequency value equal to the value of the final frequency will be given. In total, this signal will last 11 s.

### 2.2. The Estimation Algorithm of the Phasor Parameters

A new algorithm for determining the total equivalent error for each of the fifteen analyzed methods, in accordance with the procedure described in [4], was applied in the studies. The equivalent error algorithm proposed by the authors aims to determine, for each of the six synchrophasor errors (TVE, FE, RFE, RT_TVE, DT, OV) obtained according to the procedure explained in [4], the equivalent synchrophasor error (*TVE_z_*, *FE_z_*, *RFE_z_*, *RT_TVE_z_*, *DT_z_*, *OV_z_*) for each of the fifteen tested methods. Based on the obtained results, the total equivalent error is determined, indicating the method that results in the smallest total equivalent error (Er_tot), defined in Section 3.

The algorithm can be used in class P and class M devices, because it meets the requirements imposed by applicable standards [5,6,7].

Testing of the algorithm is conducted by means of computer simulations using the Matlab program (version R2024a), and its aim is to calculate measurement errors which are compared with permissible errors and on this basis the equivalent errors are determined.

### 2.3. The Phasor Magnitude and Phase Angle Estimation Algorithms

With respect to the guidelines set out in the applicable standards [5,6,7], simulations for static and dynamic signals were performed. For the synchrophasor frequency estimation, the zero-crossing method was used (described in [3,4]) as one of the most frequently considered algorithms in terms of its calculation simplicity and accuracy.

Based on the literature analysis, the authors presented various measurement algorithms used for amplitude and phase estimation of the synchrophasor: Orthogonal Components [19] (denoted as method 1), Fast Fourier Transform (DFT) [12,33] (method 2), Orthogonal Components with a single delay equal 1 [19] (method 3), Orthogonal Components with a single delay equal 64 [19] (method 4), Orthogonal Components with a double delay equal 1 [19] (method 5), Orthogonal Components with a double delay equal 21 [19] (method 6), Correlation with sine/cosine functions [19] (method 7), Convolution with orthogonal functions [19] (method 8), Least Square Method [1,2,3,4,34] (method 9), Quadrature Filter [5,6,7] (method 10), DFT method for non-nominal frequency [12] (method 11), Phase Locked Loop [35] (method 12), and methods based on artificial neural networks for estimating the initial amplitude of the synchrophasor: RBF [1,2,3,4,11,32,36,37] (method 13), FNN [32,38] (method 14), and LNN [32,38] (method 15). These methods met all requirements regarding the accuracy of signal magnitude identification in the assumed range of frequency, phase, and size changes.

For each of the methods analyzed in the article, allowing with sufficient accuracy the magnitude, phase, and frequency of the synchrophasor, an algorithm was used to introduce corrections to the results of the initial estimation of the magnitude and phase (described in [4]).

For the purposes of testing in accordance with [5,6,7], the linear phase FIR filter was used, which introduces a constant group delay, so that the difference between the input and output signals is linearly related to the frequency of the input signal. In order to compensate the filter delay, appropriate correction factors [4] were implemented for the estimated output signal phase and compensation for the magnitude estimation obtained as a result of using ANN methods. In the case of amplitude, piecewise linear approximation with a step of 1 Hz was implemented.

The first ANN method—the RBF method, presented in [3,4,11]—is universally applicable in many applications due to its quick learning process, correct generalization of data, and relatively simple network structure [36,37].

The second ANN method—the Feedforward Neural Network (method 14)—is an example of a network which consists of multiple layers, where the first of these has a connection from the network input, each subsequent layer is a connected from the previous layer, and the last layer generates the network’s output. The Feedforward Neural Network can be used for input to output mapping, but a more specialized feedforward network can found applicable in fitting and pattern recognition.

The third ANN method—the Linear Neural Network (method 15)—is a type of network which characterizes the linear transfer function; therefore, their output can be of any value. Most commonly is applicable to solve problems described linearly, because a neuron can be trained by linear approximation of a non-linear function [38]. The advantage of this network is the fact that the information obtained at the output is close to the expected goal.

The neural networks analyzed herein underwent the training process for the same signal values: constant angle values (equal to 0), frequency (50 Hz), and a magnitude varying from 0.1 pu to 2.0 pu, with a step of 0.1 pu.

In the tested RBF, FFN, and LNN networks, samples from the neighborhood of the extreme learning signal were used as the learning function. Then, after verifying whether the above conditions were met, network training was carried out. The number of the tests performed allows us to conclude that each of the three tested ANN types provide adequate accuracy. Additionally, it can be noticed that even when the ANN network has the angle and frequency values unchanged during training, the estimation for the input signal magnitudes with any frequency, amplitude, or phase parameters is correct. An important aspect is to retain as many samples as were in the learning process, which are from the learning signal neighborhood and are extreme for each tested input signal.

An additional condition that each of the three tested ANN methods meets is the use of 128 samples, which is equivalent to no more than half of each measurement window. However, the number of samples from the previous window is 30 for the analyzed functions with a frequency smaller than 50 Hz.

For the purposes of conducting appropriate tests, it was assumed that the reporting frequency will always be $F_{S}=50\;frames/s$. This means that the estimated parameters of the synchronizer should be obtained from the signal with a maximum length of 0.02 s regardless of the current network frequency. One waveform period is analyzed for a network frequency of 50 Hz or above and for a network frequency of less than 50 Hz the result should be obtained by analyzing less than one mileage period. Tests of algorithms for processing instantaneous values of current or voltage for the synchrophaser parameters and subsequent verification were carried out only with the help of computer simulations in the Matlab application [32].

When choosing the above quoted methods, the possibility of their implementation in a real power system was guided. The selected methods represent various families of algorithms in the time domain but have not been tested due to the property of amplifying noise or interference other than those described in the standard [5,6,7]. In the article, the phasor errors, TVE, FE, RFE, OV, DT, RT, for all 15 methods were determined in accordance with the guidelines in the IEEE C37.118.1 standard. The actual phasor values may differ from those obtained from PMU measurements in both amplitude and phase.

## 3. Analysis of the Results for TVE Value

**Figure 1:**
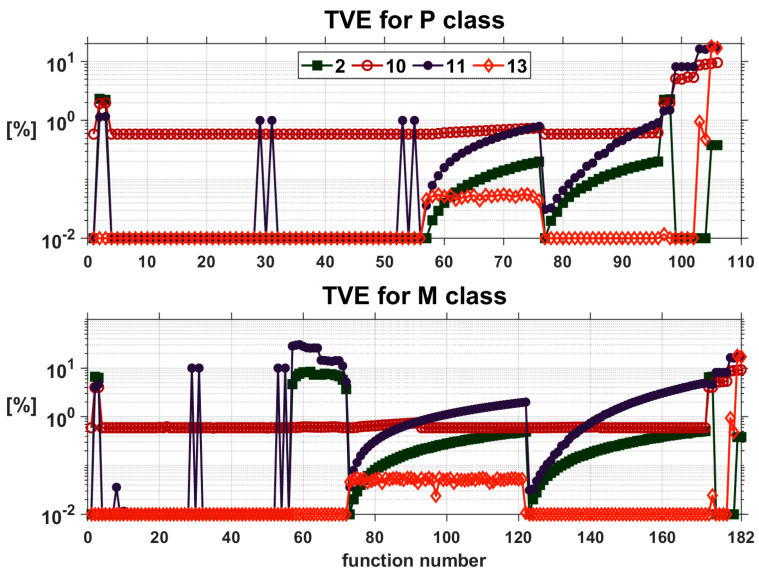
The TVE graphs for subsequent functions.

The analyses considered 15 methods of estimating the synchrophaser parameters, and for each method, six error values defined in the respective standard are determined for 106 (class P) or 182 (class M) test functions. Analyzing such a large number of results in order to choose the best method is very difficult. As an example, the TVE value results for testing functions are presented for class P and class M (Figure 1).

For each class, four methods with the smallest TVE values were selected, i.e., three classic methods and one method based on neural networks. Because the results obtained for method 13 and method 15 are practically the same, method 13 (as the first) was selected for analysis.

From the comparison of the obtained results presented in Figure 1, it can be concluded that the smallest TVE errors for all testing functions described in [5,6,7] are provided by method 13 (RBF). For only functions with a step phase change of ±π/10 and with a step occurring in the middle of the measurement window (denoted as 105 to 106 for the P class and functions 181 to 182 for the M class in Figure 1), the errors obtained by this method are large (amounting to respectively: 17.9192 and 16.9530 for both classes). From classic methods presented in this article and denoted as 1 to 12, the lowest TVE errors were obtained using method 2.

It is difficult to analyze the results for each of the 15 methods containing all tested functions and six types of estimation errors, TVE, FE, RFE, RT-TVE, DT, and OV, for each class (P and M); therefore, a new form of one error is defined, called an equivalent error.

The definition of equivalent error for the TVE, FE, and RFE values for all functions except functions with a step magnitude or functions with a step phase, described in [5,6,7], are expressed in the following formulas:

(1) $${TVE}_{z}=\sum_{j=1}^{\begin{array}{c} 174\\ 98 \end{array}}\left(\frac{{TVE}_{j}-{TVE}_{dj}}{{TVE}_{dj}}{RT-TVE}_{j}\right),$$

where ${TVE}_{j}$ is the error TVE for the jth function, ${TVE}_{dj}$ is the permissible error values TVE for the *j*th function, and ${RT-TVE}_{j}$ is the duration of magnitude, e.g., TVE, for the *j*th function above the permissible value.

(2) $${FE}_{z}=\sum_{j=1}^{\begin{array}{c} 174\\ 98 \end{array}}\left(\frac{{FE}_{j}-{FE}_{dj}}{{FE}_{dj}}{RT-FE}_{j}\right),$$

where ${FE}_{j}$ is the error FE for the jth function, ${FE}_{dj}$ is the permissible error values FE for the *j*th function, and ${RT-FE}_{j}$ is the duration of magnitude, e.g., FE, for the *j*th function above the permissible value.

(3) $${RFE}_{z}=\sum_{j=1}^{\begin{array}{c} 174\\ 98 \end{array}}\left(\frac{{RFE}_{j}-{RFE}_{dj}}{{RFE}_{dj}}{RT-RFE}_{j}\right),$$

where $R{FE}_{j}$ is the error RFE for the jth function, ${RFE}_{dj}$ is the permissible error values RFE for the jth function, and ${RT-RFE}_{j}$ is the duration of magnitude, e.g., RFE, for the *j*th function above the permissible value.

Based on the determination of the above equivalent errors, ${TVE}_{z}$ (Equation (1)), ${FE}_{z}$ (Equation (2)), and ${RFE}_{z}$ (Equation (3)), a total equivalent error ${Er}_{1}$ (Equation (4)) has been determined for each method to clearly indicate which method generates the smallest errors

(4) $${Er}_{1}=\sum_{\begin{array}{c} j \end{array}}{TVE}_{z}+{FE}_{z}+{RFE}_{z},$$

A similar procedure for determining equivalent errors for RT-TVE, DT, and OV errors defined by the standard [5,6,7] for functions with a step magnitude or functions with a step phase was used for all the considered methods:

(5) $${RT-TVE}_{z}=\sum_{\begin{array}{c} j=175\\ j=99 \end{array}}^{\begin{array}{c} 182\\ 106 \end{array}}\left(\frac{{TVE}_{j}-{TVE}_{dj}}{{TVE}_{dj}}{RT-TVE}_{j}\right),$$

(6) $${DT}_{z}=\sum_{\begin{array}{c} j=175\\ j=99 \end{array}}^{\begin{array}{c} 182\\ 106 \end{array}}{DT}_{j},$$

where ${DT}_{j}$ is the value DT for the *j*th function.

(7) $${OV}_{z}=\sum_{\begin{array}{c} j=175\\ j=99 \end{array}}^{\begin{array}{c} 182\\ 106 \end{array}}\left(\frac{{OV}_{j}-A_{j}}{A_{j}}{RT-OV}_{j}\right),$$

where ${OV}_{j}$ is the value OV for the *j*th function, $A_{j}$ is the maximum unit amplitude, which is 1.1 for overshoot or 0.9 for undershoot, the amplitude of the angle is ±π/18, after step for the jth function, and ${RT-OV}_{j}$ is the time OV jth function more than the value $A_{j}$.

For ${RT-TVE}_{z}$ (Equation (5)), ${DT}_{z}$ (Equation (6)), and ${OV}_{z}$ (Equation (7)) equivalent errors, the total equivalent error ${Er}_{2}$ (Equation (8)) has the form

(8) $${Er}_{2}=\sum_{\begin{array}{c} j \end{array}}{RT-TVE}_{z}+{DT}_{z}+{OV}_{z},$$

Based on ${Er}_{1}$ (Equation (4)) and ${Er}_{2}$ (Equation (8)) errors, a total equivalent error ${Er}_{tot}$ (Equation (9)) was determined for all functions specified in [5,6,7] and each method individually:

(9) $${Er}_{tot}=\sum_{\begin{array}{c} j \end{array}}{Er}_{1}+{Er}_{2}$$

## 4. Equivalent Error Results

Three equivalent errors: ${TVE}_{z}$ (Equation (1)), ${FE}_{z}$ (Equation (2)), and ${RFE}_{z}$ (Equation (3)), are identified for each of the 15 methods tested for all testing functions, excluding functions with a step magnitude or functions with a step phase. The results are presented in Table 1 for both class P and M.

Based on the above results, it can be concluded that for both class P and class M, the smallest ${TVE}_{z}$, ${FE}_{z}$, and ${RFE}_{z}$ errors were obtained for methods 13, 14, and 15, and the largest were obtained for method 12. Additionally, the ${TVE}_{z}$, ${FE}_{z}$, and ${RFE}_{z}$ results for class M are several times higher compared to class P for classical methods (from 1 to 12). The results for methods 13, 14, and 15 remain unchanged.

For functions with a step magnitude or functions with a step phase, the results obtained by 15 methods tested for *RT* − *TVE_z_* (Equation (5)), ${DT}_{z}$ (Equation (6)), and ${OV}_{z}$ (Equation (7)) are shown in Table 2 for both classes. An analysis of the results obtained indicates that the lowest values of replacement errors are obtained using method 2 and methods 13, 14, and 15 for both classes. The results of replacement errors obtained for classes P and M are very similar.

Because any type of disturbance may appear in the power grid, the total equivalent error Er_tot (Table 3) was defined for all 106 functions of class P and 182 functions of class M for each method. Additionally, the results of the Er_1 (for functions 1–98 for P class and 1–174 for M class) and Er_2 (for functions 99–106 for P class and 175–182 for M class) are presented in Table 3 presented for each method. Analyzing the obtained results, only neural network methods 13, 14, and 15, to a small extent, are susceptible to disturbances in the form of a step phase (described in [5,6,7]) that may appear in the power system.

## 5. Discussion and Conclusions

The paper presents a comparison of twelve methods, known from the literature, used in the estimation of the synchrophasor magnitude, phase, and frequency by PMU devices of the WAMS, with three methods based on artificial neural networks. In total, 15 different methods of synchrophasor parameter estimation were considered. All the calculations were carried out using the Matlab application. In order to indicate the usefulness of the method developed by the authors, simulations were performed for all testing functions in relation to disturbances defined in the applicable standards. This resulted in 106 testing functions for class P and 182 for class M. As a result, 25,920 error values were obtained for the selected functions, which did not allow for an unambiguous indication of the best method. Hence, it was decided to define own substitute errors for each error type, which reduced the number of errors to six. Finally, the optimal solution turned out to be the author’s equivalent error method.

The research showed that the proposed method is characterized by the effectiveness, simplicity of the calculation process, accuracy, and acceptable speed of obtaining the answer. In order to indicate the potential of the equivalent error method, appropriate simulations were performed, and the results for the *TVE_z_*, *FE_z_*, and *RFE_z_* values are presented in Table 1, while the results for the *RT-TVE_z_*, *DT_z_*, and *OV_z_* values are presented in Table 2. In order to indicate the method generating the smallest error for the obtained results, the error was divided into the equivalent error Er_1 (the sum of errors *TVE_z_*, *DT_z_*, and *OV_z_*) and the equivalent error Er_2, which is the sum of errors *RT-TVE_z_*, *DT_z_*, and *OV_z_*. Based on these two errors, the total equivalent error Er_tot was determined for each method, and the results are presented in Table 3. The effectiveness of the solutions obtained using the equivalent error method for functions excluding those functions with a step magnitude or functions with a step phase is optimal in terms of methods 13, 14, and 15, as the methods generating the smallest errors, and method 12, generating the highest error values (Table 1 and Table 3). For functions with a step magnitude or functions with a step phase, the interpretation of the results in Table 2 and Table 3 indicates method 2 as generating the lowest error values. For this group of functions, methods 13, 14, and 15 provide higher error values compared to method 2, which only proves their sensitivity to this type of disturbance, but the values of equivalent errors obtained by them do not exceed 1%.

Defining the total equivalent error (Er_tot) was intended to uniquely indicate, among the 15 methods tested, the method (or group of methods) that provides the lowest error value. The analysis of the results presented in Table 3 clearly indicates that only methods 13, 14, and 15 generate a total equivalent error not exceeding 1%. Also, the differences in Er_1, Er_2, and Er_tot obtained using the methods based on artificial neural networks are negligibly small.

Thus, the form of the total equivalent error proposed in the paper may be stated to be successfully used for the following applications:

- In PMU devices applied in the power system (due to the reliability and precision of the results of the analysis of the measurements of network parameters, mainly current and voltage) for the purposes of developing protection technologies and control processes; in the optimization of the safety margin of system operation; or in the estimation of system operation or protection algorithms in the transmission networks,
- As a criterion for selecting a method that would work equally well for measurements made at the beginning and at the end of a long line, because it eliminates higher harmonics well from the input signals and estimates the amplitude and phase parameters with sufficient accuracy in the case of functions with a magnitude modulation and the frequency changing in a specific range, or in the case of the functions with a phase modulation and the frequency changing in a specific range, gives good responses for any signal starting angle.

On the basis of the obtained values of the total equivalent error, it can be concluded that the smallest total equivalent error for the M and P class functions was obtained for algorithms based on ANN—the value of the total equivalent error was the same for all the three methods analyzed. Thus, each of them is similarly useful in estimating the synchrophasor parameters used in the PMU devices of the WAMS.

## Figures and Tables

**Table 1:** Equivalent error values for TVE, FE, RFE values.

Method Number	Equivalent Error					
	P Class			M Class		
	TVE_z_	FE_z_	RFE_z_	TVE_z_	FE_z_	RFE_z_
1	8.914	44.68	0.3348	427.9	1617	3.046
2	7.562	44.68	0.3348	168.7	1617	3.046
3	6.451	44.68	0.3348	421.4	1617	3.046
4	1.329	44.68	0.3348	383.0	1617	3.046
5	6.480	44.68	0.3348	422.0	1617	3.046
6	6.090	44.68	0.3348	427.0	1617	3.046
7	0.9379	44.68	0.3348	381.0	1617	3.046
8	0.9460	44.68	0.3348	381.2	1617	3.046
9	40.30	44.68	0.3348	629.5	1617	3.046
10	8.164	15.128	15.31	43.15	3.215	62.34
11	0.4843	39.19	0.6746	306.7	2849	8.223
12	574.3	46.13	10.76	2743	331.8	74.69
13	0.0000	0.0000	0.0000	0.0000	0.0000	0.0000
14	0.0000	0.0000	0.0000	0.0000	0.0000	0.0000
15	0.0000	0.0000	0.0000	0.0000	0.0000	0.0000

**Table 2:** Equivalent error values for RT-TVE, DT, and OV values.

Method Number	Equivalent Error					
	P Class			M Class		
	${RT-TVE}_{z}$	${DT}_{z}$	${OV}_{z}$	${RT-TVE}_{z}$	${DT}_{z}$	${OV}_{z}$
1	9.041	0.3347	1.090	9.041	0.3347	1.090
2	0.0000	0.08000	0.0000	0.0000	0.08000	0.0000
3	8.473	0.3346	1.044	8.473	0.3346	1.044
4	8.626	0.3346	1.061284	8.626	0.3346	1.061
5	8.475	0.33467	1.044366	8.475	0.3346	1.044
6	8.551	0.3347	1.053475	8.551	0.3347	1.053
7	8.860	0.3364	1.090922	8.860	0.3364	1.091
8	8.860	0.3364	1.172316	8.860	0.3364	1.172
9	8.860	0.3377	1.069	8.858	0.3377	1.069
10	1.310	0.1681	0.0000	2.819	0.7762	0.0377
11	4.076	0.0008	0.4361	4.076	0.0008	0.4361
12	118.0	0.0008	3.877	118.0	0.0008	3.877
13	0.6574	0.1995	0.02048	0.6574	0.1995	0.02048
14	0.6592	0.1995	0.02048	0.6592	0.1995	0.02048
15	0.6574	0.1995	0.0205	0.6574	0.1995	0.0205

**Table 3:** Value equivalent errors for Er_1, Er_2, and Er_tot for the P and M class of the PMU devices.

Method Number	Equivalent Error					
	P Class			M Class		
	Er_1	Er_2	Er_tot	Er_1	Er_2	Er_tot
1	53.93	10.47	64.39	2048	10.47	2059
2	52.57	0.0800	52.65	1789	0.0800	1749
3	51.46	9.852	61.32	2042	9.852	2051
4	46.34	10.02	56.36	2003	10.02	2013
5	51.49	9.854	61.35	2042	9.854	2052
6	51.10	9.939	61.04	2047	9.939	2057
7	45.95	10.29	56.24	2001	10.29	2012
8	45.96	10.37	56.33	2001	10.37	2012
9	85.31	10.27	95.57	2250	10.27	2260
10	38.59	1.479	40.07	108.7	3.632	112.3
11	40.35	4.513	44.86	3164	4.513	3168
12	631.2	121.9	753.1	3149	121.9	3271
13	0.0000	0.8774	0.8774	0.0000	0.8774	0.8774
14	0.0000	0.8792	0.8792	0.0000	0.8792	0.8792
15	0.0000	0.8774	0.8774	0.0000	0.8774	0.8774

## Data Availability

The data are contained within the article.
